# Micro-RNA 10a Is Increased in Feline T Regulatory Cells and Increases Foxp3 Protein Expression Following In Vitro Transfection

**DOI:** 10.3390/vetsci4010012

**Published:** 2017-02-21

**Authors:** Yan Wang, Mukta Nag, Joanne L. Tuohy, Jonathan E. Fogle

**Affiliations:** North Carolina State University College of Veterinary Medicine, Department of Population Health and Pathobiology and Comparative Biomedical Sciences Graduate Program (Immunology), 1060 William Moore Drive, Raleigh, NC 27607, USA; ywang102@email.unc.edu (Y.W.); mnag@ncsu.edu (M.N.); jltuohy@ncsu.edu (J.L.T.)

**Keywords:** T regulatory cell, feline immunodeficiency virus, microRNA, Foxp3

## Abstract

CD4^+^CD25^+^Foxp3^+^ T regulatory (Treg) cells are activated during the course of lentiviral infection and exhibit heightened suppressor function when compared to Treg cells from uninfected controls. Foxp3 is essential to Treg cell function and multiple studies have documented that lentivirus-activated Treg cells exhibit heightened Foxp3 expression when compared to Treg cells from uninfected controls. Our hypothesis was that lentivirus-induced micro-RNAs (miRNAs) contribute to heightened Treg cell suppressor function by stabilizing Foxp3 expression. We demonstrated that CD4^+^CD25^+^ T cells from both feline immunodeficiency virus infected (FIV^+^) cats and uninfected control cats exhibit increased miRNA 10a and 21 levels compared to autologous CD4^+^CD25^−^ T cells but there was no difference in the levels of these miRNAs when Treg cells from FIV^+^ cats were compared to Treg cells from uninfected controls. Further, there was no increase in Foxp3 mRNA following transfection of miRNA 10a or 21 into a feline cell line. However, transfection with miRNA 10a resulted in increased Foxp3 protein expression.

## 1. Introduction

T regulatory (Treg) cells, first identified as a subpopulation of CD4^+^ T cells expressing the IL2α receptor (CD25), play a major role in suppressing autoimmune T cell responses [1,2]. Subsequent studies revealed that they are also important in controlling excessive T cell responses to microbial infection, helping to control “collateral damage” associated with unchecked inflammation [3,4]. Further, it has been clearly demonstrated that Treg cells are activated during the course of lentiviral infection and exhibit heightened suppressor function when compared to Treg cells from uninfected controls [5,6,7,8]. The forkhead transcription factor Foxp3, is essential to Treg cell development and function [9,10]. Treg cell Foxp3 expression is normally under tight epigenetic (DNA methylation and demethylation) and post-transcriptional (micro-RNA, miRNA) control. For example, many investigations have clearly demonstrated that demethylation of the promoter region and the Treg-specific demethylated region (TSDR) in the Foxp3 gene lead to sustained, high levels of Foxp3 messenger RNA (mRNA) [11,12]. Further, multiple studies have documented that lentivirus-activated Treg cells exhibit heightened Foxp3 expression when compared to controls [5,6,7,13,14]. Collectively, these results suggest that lentiviral infection contributes to stable Foxp3 expression either directly through virus-specific mechanisms or indirectly via the propagation of chronic inflammation. Using the FIV model for AIDS lentiviral persistence, the focus of the study reported here was to demonstrate that miRNA modulation contributes to stable Foxp3 expression in lentivirus-activated Treg cells. 

MiRNAs play an essential role in T cell development and function, including the Treg cell subset (reviewed in [15]). MiRNAs are short (19–24 nucleotides), non-coding RNAs that are essential for regulating mRNA levels. In general, miRNAs recognize the 3′ untranslated region (3′UTR) of an mRNA sequence and binding of the miRNA to the mRNA 3′UTR leads to disruption of translation or degradation of the mRNA [16,17]. Although miRNAs typically contribute to mRNA degradation, thereby reducing translation and subsequent protein levels, there is evidence that miRNAs can also contribute to stabilization of translation complexes and increased protein production [18,19,20]. Therefore, we asked if there was differential miRNA expression in Treg cells compared to CD4^+^CD25^−^ T helper cells and in lentivirus activated Treg cells when compared to Treg cells from healthy controls. Our hypothesis was that virally-induced miRNAs stabilize Foxp3 expression in activated Treg cells from FIV^+^ cats. Current findings suggest multiple miRNAs 10a, 21, 24, 31, 95, 125, 126, and 155 modulate Treg cell development and function [21,22]. Here, we demonstrate that miRNAs 10a and 21 were expressed at higher levels in feline Treg cells. Transfection of miRNAs 10a and 21 into a feline lymphocyte cell line did not alter Foxp3 mRNA levels. However, transfection of miRNA 10a into a feline lymphocyte cell line led to increased Foxp3 protein expression. 

## 2. Experimental (Materials and Methods)

***Mya-1 Cell Culture:*** Feline Mya-1 cells are a CD4^+^ feline T cell line. Mya-1 feline T cells were cultured in Roswell Park Memorial Institute (RPMI) 1640 medium with 2 mM l-glutamine adjusted to contain 1.5 g/L sodium bicarbonate, 4.5 g/L glucose, 10 mM HEPES, and 1.0 mM sodium pyruvate and supplemented with 0.05 mM 2-mercaptoethanol, 100 units/mL recombinant human IL2 (R&D Cat# 202-IL-010), 10% fetal bovine serum and 1% penicillin and streptomycin. Cultures were maintained by the addition of fresh medium or replacement of medium. The cells were grown at 37 °C in a humidified atmosphere containing 7% CO_2_. 

**Animals and Infection:** Specific pathogen-free cats were obtained from Liberty Labs (Liberty Corners, NJ, USA) and housed at the Laboratory Animal Resource Facility at the College of Veterinary Medicine, North Carolina State University. Age matched cats were randomly assigned to either a sham-infected group (FIV^−^ control) or FIV infected group. The sham-infected group was injected with the same volume of cell free tissue culture medium as was used to culture the virus for FIV infected cats. The FIV^+^ cats were infected with 10^5^ TCID_50_ of FIV^−^ NCSU_1_ as described previously [5,8,23]. The use of cats for the studies presented here was approved under Institutional Animal Care and Use Committee (IACUC) guidelines. 

***Isolation of T-cell populations:*** Peripheral blood mononuclear cells (**PBMCs**) from FIV^+^ or FIV^−^ cats were surface stained with CD4-APC and CD25-FITC and sorted into CD4^+^CD25^−^ and CD4^+^CD25^+^ T cell populations (>95% purity) as previously reported [24,25]. 

***RNA Extraction, Reverse Transcription (RT) and Real-time PCR Quantification:*** Total RNA was extracted from cells using PureLink RNA Micro Kit (Life Technologies, Carlsbad, CA, USA). The concentration was quantified using a Nano Drop Spectrophotometer. TaqMan miRNA assays (Life technologies) were used to quantify mature miRNA expression. RNU6B was used as an endogenous small RNA control for miRNA expression studies. RT PCR was performed for miRNA using 10 ng of total RNA, 100 mM dNTP, 50 U MultiScribe reverse transcriptase, 20 U RNase inhibitor, and 50 nM of gene-specific RT primer samples using the TaqMan MicroRNA Reverse Transcription kit. The 15 μL reactions were incubated for 30 min at 16 °C, 30 min at 42 °C, and 5 min at 85 °C to inactivate the reverse transcriptase. Real-time PCR (1.33 μL of RT product, 10 μL TaqMan microRNA master mix, and 1 μL TaqMan Small RNA Assay primers and 7.67 μL Nuclease-free water) were run in triplicates at 95 °C for 10 min, followed by 40 cycles at 95 °C for 15 s and 60 °C for 1 min by using LightCycler480 (Roche Diagnostics Corporation, Indianapolis, IN, USA). miRNA expression was reported using a ΔΔCt ratio using the miRNA of interest, compared to RNU6B. 

***miRNA Transfection:*** BLOCK-iT Alexa Fluor Red Fluorescent Control (20 μM, ThermoFisher) was used to optimize transfection condition in Mya-1 cells. Lipofectamine RNAiMAX Reagent (ThermoFisher) 3 μL, 6 μL or 8 μL and BLOCK-iT Alexa Fluor Red Fluorescent Control 50 pmol, 100 pmol and 200 pmol were used as a delivery system. The 100 μM miRNA (sequence mimics) stock solution was diluted to 10 μM for immediate use. Stocks were stored at −20 °C in a non-frost-free freezer until use. Lipofectamine RNAiMAX Reagent 6 μL and miRNA 50 pM, 100 pM and 200 pM were used. Mya-1 cells were seeded at 2.0 × 10^5^/well in 24-well plate with full cell culture medium without penicillin and streptomycin. Then, 50 μL mixed reagents of Lipofectamine RNAiMAX Reagent with either BLOCK-iT Alexa Fluor^®^ Red Fluorescent Control or miRNA were added to each well. Cells were incubated at 37 °C in a humidified atmosphere containing 7% CO_2_ for 24 h to 48 h. After incubation, cells were harvested and stained with 4′,6-Diamidino-2-Phenylindole, Dihydrochloride (DAPI) for 5 min and washed. The samples were assessed by flow cytometry for the percentage of live/dead and red fluorescent protein (RFP) positive cells. 

***Western blot analysis:*** A total number of 1 × 10^6^ cells were lysed in ice-cold Nonidet P-40 (NP-40; Sigma, St. Louis, MO, USA) lysis buffer (0.5% NP-40, 50 mM Tris–HCl pH 8.0, 150 mM NaCl, 2 mM EDTA) for 30 min, then nuclei and cell debris were removed by centrifugation at 12,000 rpm for 10 min at 4 °C. Sodium dodecyl sulfate (SDS) gel-loading buffer was then added to the supernatant and the samples were denatured at 95 °C for 5 min. Protein concentrations in cell extracts were quantified prior to addition of the loading buffer with the Micro BCA Protein Assay Kit (Thermo Science, Rockford, IL, USA). Proteins (40 μg) were electrophoretically separated on a 4%–12% Bis-Tris Protein Gels, 1.5 mm (Life technologies) and electro-blotted onto a Polyvinylidene difluoride (PVDF) membrane (ThermoFisher, Waltham, MA, USA). For protein detection, the blots were probed with either a rabbit monoclonal anti-Foxp3 antibody (1:500 dilution; GeneTex, Irvine, CA, USA) or anti-beta-actin antibody (1:5000 dilution; GeneTex). To check for equal loading and transfer, the membranes were reprobed with a mouse IgG monoclonal anti-β-actin antibody (1:5000 dilution; GeneTex). Anti-mouse (Life Technologies) or anti-rabbit antibody (1:5000 dilution, Santa Cruz, CA, USA) were used as the secondary antibody. The membranes were subjected to enhanced chemiluminescence (Amersham Life Sciences) and autoradiography. 

**Figure 1:**
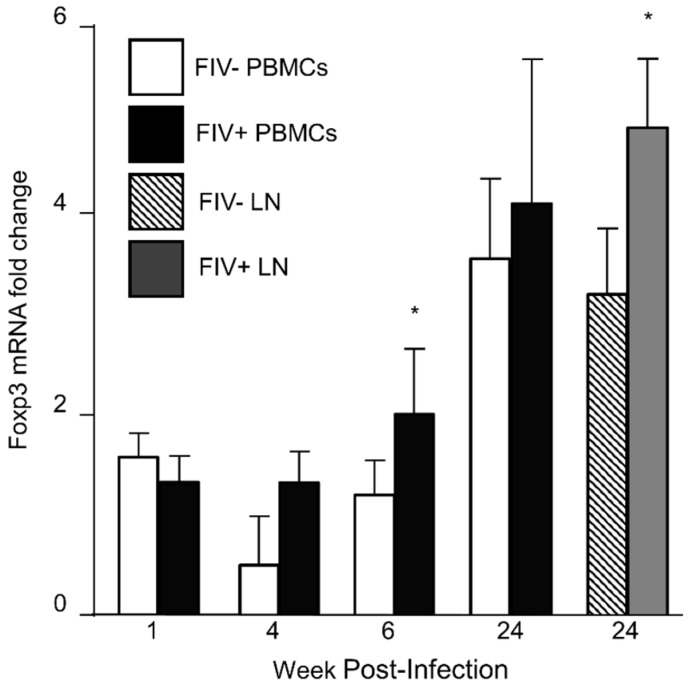
Assessment of CD4^+^CD25^+^ Foxp3 mRNA from the peripheral blood and lymph node of uninfected control cats and FIV^+^ cats. CD4^+^CD25^+^ T cells from uninfected control cats and FIV^+^ cats were isolated by cell sorting and assessed for Foxp3 mRNA levels by RT-qPCR. Foxp3 levels increased in peripheral blood mononuclear cells (PBMCs) from both FIV^−^ control cats and FIV^+^ cats over time (one way ANOVA, brackets not shown, *p* < 0.05). Foxp3 levels from peripheral blood Treg cells were increased in FIV^+^ cats (black bars) at 6 weeks post infection, compared to uninfected cats (white bars) and from the lymph node of FIV^+^ cats at 24 weeks post infection (grey bar), compared to uninfected cats (striped bar, Student’s *t*-test, * *p* < 0.05).

**Figure 2:**
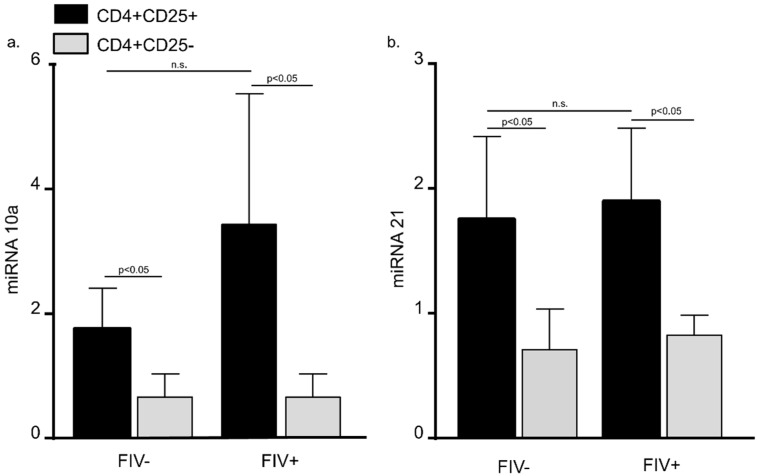
MiRNA 10a and 21 levels are increased in CD4^+^CD25^+^ lymphocytes from both FIV^−^ and FIV^+^ cats. There was no difference in miRNA 10a (**a**) and 21 (**b**) levels when peripheral blood CD4^+^CD25^+^ T cells (black bars) from FIV^+^ cats (between 6 and 12 months post infection) were compared to CD4^+^CD25^+^ cells from FIV^−^ control cats (n.s. = not significant). Both miRNA 10a and miRNA 21 were increased in CD4^+^CD25^+^ T cells when compared to autologous CD4^+^CD25^−^ T cells (grey bars) in uninfected cats and FIV^+^ cats. (Bars represent mean + S.D. for five cats, Student’s *t*-test, *p* < 0.05).

**Figure 3:**
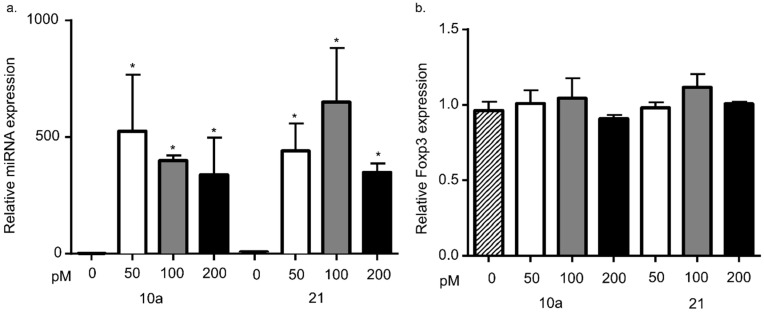
Foxp3 mRNA levels after miRNA 10a or 21 transfection. (**a**) Mya-1 cells were transfected with miRNA10a (left) and miRNA 21 (right) at increasing concentrations and the relative expression levels were assessed by RT-qPCR. The amount of miRNA expression dramatically increased after transfection at all concentrations (one-way ANOVA with post-hoc Tukey, * *p* < 0.01). (**b**) The same groups of infected cells were also assessed for Foxp3 mRNA levels between 24 and 48 h after transfection. There was no change in Foxp3 mRNA levels in any of the groups transfected with either miRNA 10 (left) or miRNA 21 (right). Bars represent the mean + S.D. for 4–5 transfection experiments.

**Figure 4:**
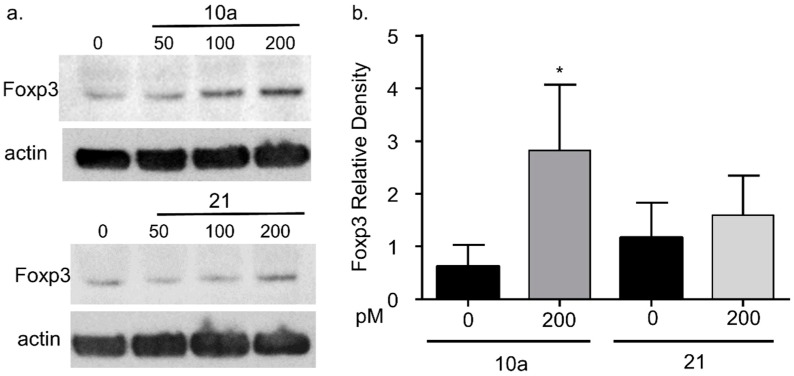
Assessment of Foxp3 protein levels following miRNA 10a and 21 transfection**.** Cells were transfected with miRNA 10a and 21 as described in Figure 3. Following transfection, intracellular Foxp3 protein expression was evaluated for miRNA 10a and 21 transfected cells (**a**) Western blot demonstrating Foxp3 protein expression following transfection with increasing amounts (0, 50, 100, 200 pM) of miRNA 10a (upper) or miRNA 21 (lower). (**b**) The relative density of Foxp3 is increased following maximal miRNA 10a transfection (200 pM, Student’s *t*-test, * *p* < 0.05) but was unchanged following miRNA 21 transfection (*p* = 0.21). Each bar represents the mean + S.D. for four separate experiments.

***Statistical Analysis:*** Statistical analysis was performed using GraphPad Prism software. For paired data (Figure 1, Figure 2, and Figure 4) a Student’s *t*-test was used. A one-way ANOVA was used to compare each time point for FIV^−^ control cats and FIV^+^ cats (intragroup, Figure 1). For multiple comparisons (Figure 3), a one-way ANOVA with a post hoc Tukey test was used. For all of these, significance was set at *p* ≤ 0.05.

## 3. Results

### 3.1. Foxp3 mRNA Is Increased in CD4^+^CD25^+^ Treg Cells during FIV Infection

Foxp3 mRNA and protein are increased in lentivirus activated Treg cells when compared to Treg cells from uninfected cats [8,26]. To document increased Foxp3 expression in Treg cells for these studies, we harvested peripheral blood Treg cells from FIV^−^ control cats and FIV^+^ cats 1 week, 4 weeks, 8 weeks and 24 weeks post infection (p.i.) and from the lymph node at 24 weeks p.i. (Figure 1). Foxp3 expression in peripheral blood Treg cells is dynamic during the acute phase of FIV infection and was higher in the peripheral blood of FIV^+^ cats at 6 weeks post infection, compared to FIV^−^ cats (Figure 1). By 24 weeks p.i., there is increased Foxp3 expression in the lymph node (LN) of FIV^+^ cats. Foxp3 also increased over time within each group (intragroup). The results reported in Figure 1 are consistent with previous studies which suggest that lentivirus-activated Treg cells exhibit higher Foxp3 mRNA and protein when compared to uninfected cats [8,25]. 

### 3.2. MiRNA 10a and 21 Expression in CD4^+^CD25^+^ and CD4^+^CD25^−^ Cats from FIV^−^ Control Cats and FIV^+^ Cats

Recent reports suggest that multiple miRNAs play an important role in regulating Treg cell Foxp3 expression function [21,22]. Database prediction software was used to identify miRNAs likely to bind to the feline Foxp3 3′UTR. Based upon recent reports and our database predictions, we focused upon miRNA 10a and 21 for the studies that follow here. Peripheral blood CD4^+^CD25^+^ and CD4^+^CD25^−^ T cells were harvested from FIV^−^ control cats and chronically infected (between 6 months and 12 months post infection) FIV^+^ cats. When CD4^+^CD25^+^ T cells from FIV^+^ cats were compared to FIV^−^ control cats, there was no difference in miRNA 10a and 21 levels (Figure 2). However, both miRNA 10a and miRNA 21 were increased in CD4^+^CD25^+^ T cells when compared to autologous CD4^+^CD25^−^ T cells in uninfected cats and FIV^+^ cats (Figure 2).

### 3.3. miRNA 10a or 21 Transfection Does Not Alter Foxp3 mRNA Levels

Based upon the findings above, we hypothesized that miRNA 10a or 21 stabilize Foxp3 expression by stabilizing Foxp3 mRNA levels within the cell. To investigate the effect of miRNA 10a and 21 on Foxp3 expression, we asked if overexpression of these miRNAs in feline Mya-1 cells (a feline CD4^+^ T cell line) would lead to increased Foxp3 expression. Basal expression of both miRNAs was measured in feline Mya-1 cells. Cell death and transfection efficiency were measured with increasing concentrations of transfection reagent and red fluorescent transfection control as described in the methods. Cell death was not altered by treatment with the transfection reagent together with the fluorescent transfection control and the maximal percentage of positively transfected cells was approximately 70%. Cells were transfected with either miRNA 10a or miRNA 21 at three different miRNA concentrations, leading to much higher miRNA expression in transfected cells (Figure 3a). Despite increased levels of miRNA 10a or miRNA 21 following transfection, Foxp3 mRNA levels remained unchanged when compared with the non-transfected control group (Figure 3b). 

### 3.4. miRNA 10a Transfection Increases Foxp3 Protein

Although Foxp3 mRNA levels appeared to be unaffected by miRNA 10a or 21 transfection, there are several reports of miRNAs modulating intracellular protein expression [18,27]. Therefore, we assessed intracellular Foxp3 protein levels following miRNA 10a or 21 transfection (Figure 4). Initial Western blot experiments demonstrated that Foxp3 exhibited variable protein expression at lower transfection levels (50 and 100 pM) for miRNA 10a (Figure 4a). Therefore, Mya-1 cells were transfected with 200 pm miRNA 10 or 21 for all subsequent experiments. Foxp3 protein was increased in Mya-1 cells following 200 pM miRNA 10a transfection but remained unchanged following 200 pM miRNA 21 transfection (Figure 4b). These results suggest that Foxp3 expression is positively regulated by miRNA10a.

## 4. Discussion

Using the FIV model, it has been previously demonstrated that FIV infection phenotypically and functionally activates CD4^+^CD25^+^Foxp3^+^ Treg cells during both the acute and chronic stages of infection and these cells are capable of inhibiting CD4^+^ and CD8^+^ T cell effector responses. Activated feline Tregs from FIV^+^ cats up-regulate CTLA4, B7.1 (CD80), B7.2 (CD86), and suppress T cell proliferation, IL2 production, and IFNγ production [5,8,13]. Further, it is clear that there is preferential replication of FIV in the CD4^+^CD25^+^ subset, both in vitro and in vivo [8,28]. These data indicate a unique relationship between lentiviral infections and T regulatory cells, in that CD4^+^CD25^+^ T regulatory cells are preferentially infected early during the course of infection, are activated early during the course of FIV infection, and are able to effectively suppress CD4^+^ effector responses in both the acute and chronic stages of FIV.

Foxp3 is essential to the development of CD4^+^CD25^+^ regulatory T cells [9,10]. Mutations in the Foxp3 gene cause scurfy in mice and immunodysregulation polyendocrinopathy enteropathy X-linked syndrome (IPEX) in humans. Scurfy and IPEX are characterized by marked immunoproliferation leading to autoimmune polyendocrinopathy and enteropathy [9]. Structurally, Foxp3 has four distinct domains: the forkhead, leucine zipper, zinc finger, and a proline rich domain at the amino terminus [29]. The forkhead domain is responsible for DNA binding at the IL2 promoter region, leading to the suppression of IL2 transcription [30]. 

Because Foxp3 is essential for Treg cell function and Treg cells exhibit heightened suppressive activity during the course of lentiviral infection, we have been exploring mechanisms that lead to sustained and heightened levels of Foxp3 within the Treg cell. Foxp3 in peripheral blood CD4^+^CD25^+^ Treg cells increased over time in both uninfected control cats and FIV^+^ cats (Figure 1). Foxp3 expression is dynamic and changes over time in growing animals as well as exhibiting temporal variability in mature animals [8,31,32,33]. Further, CD4^+^CD25^+^ Treg cells exhibited higher levels of Foxp3 mRNA from the blood and LN of FIV^+^ cats when compared to uninfected control cats (Figure 1). These findings are consistent with those previously reported [8]. How lentiviral infection contributes to heightened Treg cell function and increased Foxp3 mRNA levels is still unclear. 

Foxp3 transcription, translation, and protein function are normally tightly regulated within the cell. At the genomic level, Foxp3 transcription is controlled by other transcription factors, such as NFAT, AP-1 and Sp1 binding to the promoter region, reviewed in [34]. At the epigenetic level, Foxp3 expression is controlled by the DNA and histone methylation status and histone acetylation status at the Foxp3 promoter and transcription start site, reviewed in [35,36]. Multiple miRNAs 10a, 21, 24, 31, 95, 125, 126, and 155 are reported to modulate Treg cell development and function [21,22]. Most miRNAs regulate gene expression by binding to the mRNA 3′UTR, leading to message degradation; however, there is evidence that miRNAs can also contribute to stabilization of translation complexes and increased protein production [18,19,20].

We hypothesized that lentiviral infection may contribute to a miRNA profile that positively regulates Treg cell Foxp3 mRNA stability. Therefore, we compared the miRNA profile of peripheral blood CD4^+^CD25^+^ T cells and CD4^+^CD25^−^ T cells from FIV^−^ control cats to the miRNA profile of CD4^+^CD25^+^ T cells and CD4^+^CD25^−^ T cells from FIV^+^ cats. Ideally, miRNA 10a and 21 would be assessed in CD4^+^CD25^+^ T cells from the LN of FIV^+^ and control cats. However, at the time of this study, lymph nodes from these cats had been collected for a concurrent study. Utilizing PBMCS instead of LN cells is a limitation of this study. As shown in Figure 2, we identified increased expression of miRNA 10a and miRNA 21 in CD4^+^CD25^+^ T cells when compared to autologous CD4^+^CD25^−^ T cells, suggesting that both of these miRNAs might play a role in Foxp3 induction or stability. Although there was a trend toward higher miRNA 10a levels in CD4^+^CD25^+^ Treg cells from FIV^+^ cats when compared to FIV^−^ cats, the results were not significant. These results did not support our hypothesis that lentiviral infection alters Treg cell miRNA 10a and 21 profiles.

Next, we asked what effect the transfection of these two miRNAs might have on Foxp3 mRNA expression. MiRNA 10a is associated with Foxp3 expression and functional Treg cell suppressive activity [21,22,37]. Further, miRNA 10a expression is heightened by TGFβ/retinoic acid signaling, leading to stable Foxp3 expression in inducible Treg cells. We have previously reported that TGFβ signaling is imperative for feline Treg cell function, induction, and suppressive activity [5,8,25]. Collectively these results indicate that miRNA 10a likely plays a central role in Treg cell homeostasis and function. However, the role of miRNA 21 in Treg cell function is more complicated. A recent report suggests that miRNA 21 suppressed the expansion of Foxp3^+^ Treg cells [38]. Others have reported that increased expression of miRNA 21 is associated with increased Foxp3 mRNA and heightened Treg cell function [39,40,41]. Our hypothesis was that these miRNAs might contribute to Foxp3 mRNA stability and thus heightened Treg cell suppressor function. As demonstrated in Figure 3, neither miRNA 10a nor miRNA 21 transfection of Mya-1 cells resulted in increased Foxp3 mRNA levels. These results can be explained in a number of ways, the simplest of which is that these miRNAs do not interact with the Foxp3 transcriptome. It is also possible that our miRNA10a and miRNA21 sequences were mismatched to the feline Foxp3 3′UTR and were therefore incapable of binding this region. 

Although Foxp3 mRNA levels remained unchanged, it is possible that these miRNAs affected protein expression without changes in mRNA levels. For example, miRNAs can form miRNA–protein complexes that influence cellular protein expression and target specific mRNAs for activation or repression, based upon the stage of the cell cycle [18,42]. Therefore, we assessed Foxp3 protein expression following miRNA 10a or 21 transfection. As demonstrated in Figure 4, there was increased Foxp3 expression following miRNA 10a transfection, but this was unchanged after miRNA 21 transfection. Taken together, these results suggest that miRNA 10a is important for Foxp3 protein expression and thus Treg cell function. 

## 5. Conclusions

It is likely that miRNA 10a cooperates with other miRNAs to modulate Treg Foxp3 expression. Because Treg cells from FIV^+^ cats exhibit heightened Foxp3 expression, we hypothesize that there may be other virally-induced miRNAs, such as miRNA 24, 31, 125a, 126, and 155, that stabilize Foxp3 mRNA or protein. Investigations are currently underway to identify and to assess the role that these other miRNAs might play in Foxp3 stabilization. Once these miRNAs are identified, they may prove to be new targets to reduce Treg cell suppression and augment T cell function for HIV vaccination and intervention strategies.
